# Smartphone Application-Based Rehabilitation Program for Older Adults With Type 2 Diabetes Mellitus: Insights From the Design, Development, and Validation

**DOI:** 10.1177/19322968251365664

**Published:** 2025-08-25

**Authors:** Tina Agnes, G. Arun Maiya, Muralidhar M. Kulakarni, Sawan Ramesh Hegde, Kirthinath Ballal, Mamatha Balachandra

**Affiliations:** 1 Centre for Podiatry & Diabetic Foot Care and Research, Department of Physiotherapy, Manipal College of Health Professions, Manipal Academy of Higher Education, Manipal, India; 2 Department of Community Medicine, Kasturba Medical College, Manipal Academy of Higher Education, Manipal, India; 3 Manipal School of Information Sciences, Manipal Academy of Higher Education, Manipal, India; 4 School of Computer Engineering, Manipal Institute of Technology, Manipal Academy of Higher Education, Manipal, India

**Keywords:** aged, smartphone application, type 2 diabetes mellitus, understandability, usability, validation

## Abstract

**Background:**

Older adults facing chronic health challenges may experience significant advantages from smartphone technologies. This study aimed to design, develop, and validate a smartphone application to deliver rehabilitation programs to older adults with type 2 diabetes mellitus.

**Methods:**

This study consisted of three phases: (1) Assessment of need, (2) Design and development of the smartphone application, and (3) Evaluation of the smartphone application by content validation. The Patient Education Materials Assessment Tool for Audiovisual Materials (PEMAT-A/V) validated the smartphone application. This validation was performed through direct interaction between the validators and the system, relevant observations were identified, and suggestions were noted during and after the test.

**Results:**

The smartphone application consisted of six modules, with content delivered through photos, videos, text, and flyers. Eight experts and twenty-five older adults over 60 years and diagnosed with type 2 diabetes mellitus completed the validation. The smartphone application received a good median understandability score of over 90 from experts and over 80 by end users, and a median actionability score of 100 by experts and over 100 by end users on the PEMAT-A/V.

**Conclusion:**

This newly developed smartphone application has been validated and is now ready for evaluation to assess its effectiveness in improving the delivery of rehabilitation programs in older adults with type 2 diabetes mellitus.

## Introduction

The prevalence of Type 2 Diabetes Mellitus (T2DM) among older adults is escalating globally, primarily due to increased longevity and prolonged exposure to cardiometabolic risk factors like excessive adiposity, muscle atrophy, and diminished physical activity levels. These factors can significantly impact these older adults’ well-being and have emerged as a serious global public health concern.^1-3,4^ Self-management support is essential to enhance an individual’s capacity to live with T2DM, regardless of whether the intervention involves behavioral, educational, psychosocial, or clinical components.^5^ Apart from the current therapies incorporated into diabetes care, smartphone health applications, or mobile health (mHealth) apps, have become additional tools for diabetic self-management.^6^

In 2018, over 100,000 such applications were available to assist users in their health self-care.^7,8^ Older adults tend to have higher smartphone ownership than desktop or laptop computers. Therefore, due to their widespread use, smartphones would be the preferred technology platform if designers and healthcare professionals want to reach most older adults.^9,10^ These applications can empower older adults with self-management tools and personalized health recommendations and reduce healthcare costs by minimizing the need for in-person visits and hospitalizations. Hence, smartphone applications present a significant opportunity to enhance chronic disease management through improved access to healthcare services.^11-14^ When developing smartphone applications for sensitive domains such as healthcare, expert validation is essential to ensure the application’s features adhere to robust scientific foundations. This is crucial for safeguarding user safety and fostering user trust.^14-16^ Despite growing interest in using smartphone applications to manage diabetes and support rehabilitation, research indicates significant shortcomings in their design and development, especially for older adults in low- and middle-income countries (LMICs). Many health apps, designed primarily within the context of high-income countries, often leave LMICs underserved, frequently failing to adopt geriatric design principles and lack personalization.^17,18^ Hence, this study aimed at designing, developing, and validating the smartphone application named “DiabFitt” to deliver a rehabilitation program for older adults with T2DM.

## Methodology

**Figure 1.**
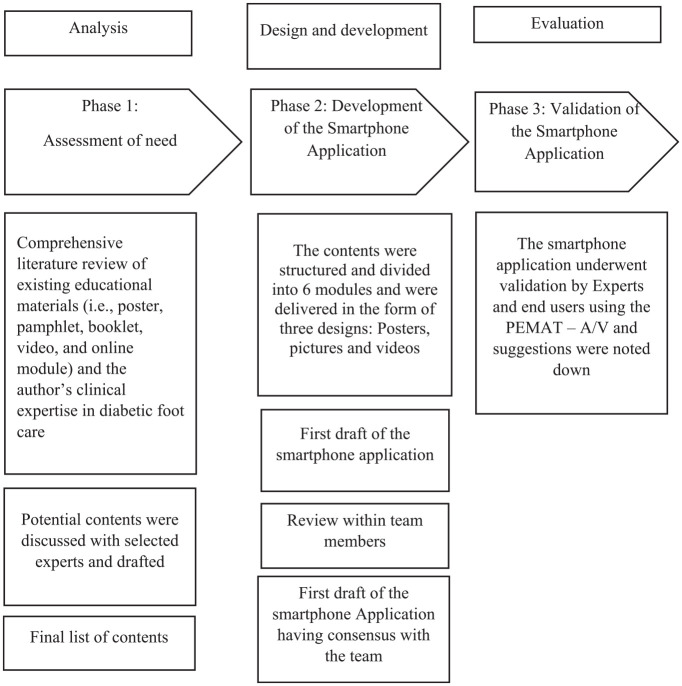
The figure illustrates the steps involved in the smartphone application’s contents.

This usability validation study is part of a larger randomized controlled trial, investigating the effectiveness of a comprehensive rehabilitation program delivered through the smartphone application to community-dwelling older adults with T2DM. The study was approved by the Kasturba Medical College and Kasturba Hospital Institutional Committee (IEC1–332–2022), and the trial has been registered under the Clinical Trials Registry—India (CTRI/2023/08/056418). The study consisted of three phases: (1) assessment of need, (2) design and development of the application (Supplemental Material), and (3) evaluation of the smartphone application (Figure 1).

### Assessment of Need

This phase focused on identifying content for the smartphone application, guided by a comprehensive literature review of existing educational resources (ie, online modules, videos, posters, pamphlets, booklets) and the author’s clinical expertise in diabetic foot care. The content was divided into six domains that formed the framework for the application’s design: Exercises, Footwear recommendations, Foot Care education, Fall prevention strategies, Lifestyle management, and Stress management (Supplemental Material No. 1). The American College of Sports Medicine Guidelines were followed for exercise prescription, which included aerobic exercise, which suggested walking because of its commonality and acceptance in the geriatric population.^19,20^ The smartphone application has been designed to deliver flexibility and strength training of major muscle groups from 0 weeks to 3 weeks, 4 weeks to 9 weeks, and 10 weeks to 12 weeks through the Application (Supplemental Material No. 2). The exercise modules within the application was designed to adapt to the unique requirements of each user. Certified physiotherapists can modify the exercises after assessing the older adults. Exercises can be simplified or altered in response to user-reported discomfort or challenges during initial trials. For the domain of footwear recommendation, Pictures of advisable footwear and what type of footwear should not be worn were uploaded to the application. Educational posters were developed for the Domains of foot Care education, fall prevention strategies, Lifestyle management, and Stress management. Domains focusing on exercise and foot care were included because diabetic foot care is a frequently neglected component of overall diabetes management in many LMICs like India; additionally, foot care is one of the most behaviorally modifiable and commonly overlooked aspects of self-care in older adults. Social customs, religious practices, and economic constraints contribute to a high prevalence of barefoot walking. At the same time, poverty and limited education often result in the use of inadequate footwear and delayed presentation of foot lesions. Diabetic foot care warrants increased attention due to its frequent neglect in standard diabetes management and its significant connection to rehabilitation services. While general diabetes care is typically included in standard healthcare protocols, foot care often receives insufficient emphasis. These domains are also directly related to mobility and quality of life.^21,22^ To ensure ease of use for older adults and to address technical integration challenges, glucose-monitoring functionalities were intentionally omitted from the smartphone application.

### Design and Development of the Smartphone Application

#### The application

Given the widespread use of smartphones, especially Android devices in India and other LMICs, we developed the application for this accessible platform. Projections suggest Android-based smartphone usage in India will surpass 90% by 2032.^23,24^ The system includes two applications that could be interconnected via the internet. One was envisioned as a web-based application for the therapist to upload and monitor the rehabilitation programs. The other was an android smartphone application that delivered exercises and patient education resources. Furthermore, the application needed to (1) be customizable regarding the content (eg, texts, pictures, videos, personalized exercise materials, and language) and (2) enable monitoring of progress by providing item-wise data following the exercise sessions. For the application, it was decided to maintain English and Kannada (the regional language of Karnataka, a southwestern Indian state where the study was conducted) as the operating languages (Supplemental Material No. 3). The participant using the application can choose the language of preference at the login time. The User interface design and application features were developed based on insights from the existing literature regarding older adults’ preferences and experiences.^25^ The program may be stored and distributed as an Android Application Package (APK) file and runs on the Kotlin platform. The application is compatible with all Android versions and processors. For data synchronization, the application will function online. The data will be protected by a password-secured server that is accessible only to the researchers and stored offline.

#### Registration and scheduling

The therapist registers the individual on the web portal, generating a five-digit one-time password for login. Exercise and patient education resources will be added based on the prescribed plan (Supplemental Material Nos. 4 and 5).

#### Flexibility

The application offers therapists flexibility to tailor exercises for older adults with T2DM, adjusting repetitions, weights, and durations. Once set, the user can view the planned exercise, which synchronizes with the application (via an internet connection) (Supplemental Material No. 6).

#### Customization and notification

It is crucial to establish exercise goals that are pertinent and significant to the individual with T2DM and fulfill their specific needs. This application enables clinicians to upload customized therapeutic resources for their clients. Specifically, the required media file [Pictures (.jpeg), Videos (MP4), or text] can be integrated into the predetermined rehabilitation template (Supplemental Material No. 7). The Notifications Enabler is designed to facilitate prescheduled or event-driven, user-friendly notifications (Supplemental Material No. 8).

#### Progress monitoring

Therapists prefer to be able to monitor physical therapy or exercise progress,^26^ and this has been made possible through the application. These provide the clinician with information to evaluate the number of training items accessed by the older adult with T2DM. The reports can be downloaded as an Excel spreadsheet. (Supplemental Material No. 9)

### Evaluation of the Smartphone Application by Content Validation

The Patient Education Materials Assessment Tool for Audiovisual Materials (PEMAT-A/V) and the think-aloud test principles were utilized to validate the application with direct interaction between the experts, end users, and the system, and notes were taken during and after the test.^27^ The PEMAT-A/V is designed to assess the understandability and actionability of patient education materials presented in an audiovisual format with a moderate to excellent interrater reliability (Kappa 0.57) and strong internal consistency (Cronbach’s α 0.87 for understandability and 0.78 for actionability), and the validity is supported by expert consensus and field testing. PEMAT-AV has been developed to be usable by laypersons and healthcare professionals without prior training to use the tool. Hence, no formal training was provided in this study; the validators received a brief explanation of how to use the smartphone application and complete the PEMAT-A/V. The PEMAT-A/V also consists of a detailed written guide on how to score the tool. The scoring comprises two domains: understandability (13 items) and actionability (4 points). Each item can be scored as Agree (1 point or Disagree/ Not applicable (0 Points). The percentage of the score is calculated as (total score/ maximum possible score) × 100, separately for each domain.^28^ Two participant groups were recruited. Experts (n = 8) and “end users” were defined as older adults (≥60 years) with T2DM (n = 25). The purposive sampling method was used to select the multidisciplinary team of experts, including four physiotherapists, one prosthetics and orthotics specialist, one health information management professional, and two engineers with expertise in smartphone application development and machine learning with at least five years of experience. Due to the exploratory nature of the study, a formal sample size calculation was not performed. Studies suggest that a sample size of 3 to 8 experts is sufficient for usability validation. This usability validation study involved 25 patients representing the smartphone application’s intended user population. This sample size was chosen based on previous research, demonstrating that pilot usability testing with 15 to 20 people reliably discovers most usability difficulties in digital health tools.^29,30^ Older adults with T2DM were recruited from the institution’s outpatient department. The inclusion criteria for the end users were (1) individuals between the ages of 60 and 75 years, (2) a diagnosis of T2DM for at least one year, (3) ownership of an Android smartphone and access to the internet, (4) able to read and understand the English language. Participants were excluded if they had any diagnosed psychiatric illness or visual impairment that could hinder their smartphone application use.

The developed smartphone application underwent a validation process by a panel of eight experts and twenty-five end users. The participants were approached, and if they consented to participate, written informed consent was obtained. They were then asked to run the application for a day, after which the PEMAT-A/V was administered to validate the smartphone application for understandability and actionability. The participants were explained the components of the PEMAT-A/V tool, and the user guide was also provided so they could understand and apply the tool effectively. Experts evaluated the smartphone application after guided navigation. The end users were provided a brief overview of the application’s features. They were allowed to independently explore the smartphone application for an hour, after which the PEMAT-A/V was provided. The methods used in this paper are low-cost, easy to implement, and provide quick, clear feedback.^31,32^

#### Statistical analysis

Data analysis was performed using Jamovi (version 2.6.26). Descriptive statistics were used to summarize demographic information. The Shapiro–Wilk test indicated that the PEMAT-AV scores were not normally distributed; therefore, understandability and actionability are presented as medians, accompanied by minimum and maximum values. The minimal score variability observed suggests a ceiling effect, indicative of a high level of agreement among raters. Given this study’s exploratory nature and the expert panel’s limited size, inferential statistical analyses and group comparisons were deemed inappropriate.

## Results

The application’s content was organized into six key areas: Exercises, Footwear Recommendations, Foot Care Education, Fall Prevention Strategies, Lifestyle Management, and Stress Management (Table 1).

**Table 1.** Overview of Smartphone Application.

Module	Mode of delivery	Description and no. number of pages of the flyers/duration of videos
*Module 1*: Exercises	Videos and step-by-step pictures of the exercises	Twenty-three exercises with three-step demonstrations in pictures and videos ranging from 5 to 15 seconds
*Module 2*: Footwear recommendations	Pictures of Footwear with a description of the footwear	*The content was divided for Males and females*^a^ Male: three pictures of recommended footwear and one picture of footwear that should be avoided Female: Two pictures of recommended footwear and one picture of footwear that should be avoided
*Module 3*: Footcare education	Posters/flyers with a description of foot care	Four posters on footcare education 1: Tips for glucose control 2: Foot changes to identify 3: Assessments for diabetic foot 4: Tips for foot care
*Module 4*: Fall prevention strategies	Posters/flyers with tips on fall prevention	One poster with tips on fall prevention
*Module 5*: Lifestyle management strategies	Posters/flyers with tips on eating healthy, the importance of physical activity, and	Two posters on lifestyle management 1. General tips on the importance of healthy eating and remaining active 2. Normal glycemic parameters
*Module 6*: Stress management	Posters/flyers with tips on managing stress	One poster on general tips for managing stress

a In India (the country where the smartphone application was developed), the footwear worn by males and females is different. Hence, different footwear was recommended.

Eight experts (mean age 31.87 ± 3.60 years; mean experience 7.37 years; SD ±3.62) and 25 end users (mean age 66.32 ± 4.12 years) validated the smartphone application using the PEMAT-A/V. Experts included physiotherapists (50%), followed by engineers (25%), prosthetists and orthotists (12.5%), and health information management professionals (12.5%) (Table 2). Among end users, 60% were male and 40% were female, with a mean T2DM duration of 10.88 ± 5.5 years (Table 3).

**Table 2.** Demographics of the Experts (N = 8).

Variables	Value
Age in years (mean ± SD)	31.87 ± 3.60
Profession—n (%)	
Physiotherapist	4 (50%)
Prosthetist and orthotist	1 (12.5%)
Health information management professional	1 (12.5%)
Engineers	2 (25%)
Years of experience in years (mean ± SD)	7.37 ± 3.62

**Table 3.** Demographics of the End Users (N = 25).

Variables	Value
Age in years (mean ± SD)	66.32 ± 4.12
Gender (%)	
• Male	15 (60%)
• Female	10 (40%)
Duration of type 2 DM in years (mean ± SD)	10.88 ± 5.5

Regarding the expert validation, all six modules had high PEMAT-A/V scores for understandability and actionability. Understandability was consistently 100%, with no variation in participant ratings. Actionability scores were also high, with a median of 100%. Modules 1 and 3 showed slight variation, ranging from 90% to 100%. Overall, experts perceived all the educational modules as highly comprehensible and actionable (Table 4).

**Table 4.** Scores of PEMAT-AV: Experts (N = 8).

Module	Understandability score (%)		Actionability score (%)	
	Median (IQR)	Min^a^, max^b^	Median (IQR)	Min^a^, max^b^
*Module 1*: Exercises	100 (0.00)	90,100	100 (0.00)	100,100
*Module 2*: Footwear recommendations	100 (0.00)	100,100	100 (0.00)	100,100
*Module 3*: Footcare education	100 (0.00)	90,100	100 (0.00)	100,100
*Module 4*: Fall prevention strategies	100 (0.00)	100,100	100 (0.00)	100,100
*Module 5*: Lifestyle management strategies	100 (0.00)	100,100	100 (0.00)	100,100
*Module 6*: Stress management	100 (0.00)	100,100	100 (0.00)	100,100

Scores of the understandability and actionability of the smartphone application by experts. IQR, interquartile range; Max, maximum score.

a Min, minimum score; Min represents the lowest percentage score obtained from all experts (n = 8).

b Max: represents the highest percentage score obtained from all the experts (n = 8).

After the end-user validation, modules 1, 3, and 4 had a median understandability score of 100% with no variation (interquartile range [IQR] = 0). Modules 2 and 5 had slightly lower medians of 90% (range 90%-100%), module 6 had a median of 100% with slightly greater variability (IQR = 20; range 80%-100%). Actionability scores were uniformly high across all modules and a consistent median and range of 100%, which indicates that the individuals with T2DM found the content easy to act upon (Table 5).

**Table 5.** Scores of PEMAT-AV: End Users (N = 25).

Module	Understandability score (%)		Actionability score (%)	
	Median (IQR)	Min^a^, max^b^	Median (IQR)	Min^a^, max^b^
*Module 1*: Exercises	100 (0.00)	100,100	100 (0.00)	100,100
*Module 2*: Footwear recommendations	90 (10)	90,100	100 (0.00)	100,100
*Module 3*: Footcare education	100 (0.00)	100,100	100 (0.00)	100,100
*Module 4*: Fall prevention strategies	100 (0.00)	100,100	100 (0.00)	100,100
*Module 5*: Lifestyle management strategies	90 (10)	90,100	100 (0.00)	100,100
*Module 6*: Stress management	100 (20)	80,100	100 (0.00)	100,100

Scores of the understandability and actionability of the smartphone application by experts. IQR, interquartile range; Max, maximum score.

a Min, minimum score. Min represents the lowest percentage score obtained from all end users (n = 25).

b Max represents the highest percentage score obtained from all the end users (n = 25).

The higher the PEMAT-A/V scores, the better the understandability and actionability. A cutoff of 70% was considered if a domain had to undergo significant changes. Since all domains in the smartphone application scored more than 80%. No significant changes were made. Only the comments given in Table 6 were incorporated into the smartphone application.

**Table 6.** Comments on the smartphone application.

Sl. No.	Module	Suggestion	Suggested and accepted modifications
1	Exercise	A sequence can be followed where foot exercises are first, then knee, then hip, so that the patient can follow a pattern	The sequence was added to the exercise order according to the exercise position
		Precautions to take before and during exercise can be added	A pop-up was added every time the individual tried to access the exercises
2	Instruction text	The text in the application can be kept in a bigger font size Font text can be dark on a light background	The font size was increased to a legible font The font color was black on a white background
3	Footwear	Add a description to the footwear	A description of footwear and the types that should and should not be worn was also added.

Comments were obtained on the module exercise, footwear description, and instruction text (Table 6).

## Discussion

T2DM continues to be a significant global health challenge worldwide, with older adults being a vulnerable population, including in low- to middle-income nations.^33^ In the present study, we successfully developed a smartphone application for older adults with T2DM, which was 1. Developed by experts 2. Easy understandability and actionability are delivered through videos, pictures, and posters. One of the focuses of this study was to examine the usability and understandability. Consequently, we do not present intervention outcomes in this study. Despite the growing use of smartphone devices in older adults, a few studies have considered the development and validation of a smartphone application by experts and end users, along with their perspectives.

The process commences with identifying the target population and their specific needs, paving the path for a user-centered approach in the design that integrates expert advice with end-user feedback, which is essential for ensuring the usability and effectiveness of the smartphone application.^34^ The factors considered during the design were *simplification*—the cognitive declines typically accompanying aging augment the difficulties that older adults face in comprehending and retaining information about how to utilize intricate systems. The goal is to reduce complexity for this user group.^35^ *Enlarging the size and spacing between interactive controls*—touchscreens require larger touch targets than desktop or web-based systems accessed with a mouse. This is because fingers are much larger than a mouse pointer and have lower precision when selecting targets than mouse clicking.^25^ *Use of videos along with text and photos of the exercise—*studies where older adults used smartphone applications revealed that participants were tired easily when presented with lengthy textual instructions, supporting the recommendation to prioritize video tutorials over written guidance.^36,37^ Our study used *large fonts and high contrast between foreground and background color.* This recommendation primarily benefits users with visual impairments but can also potentially benefit a wide range of individuals, such as those using screens with low brightness or experiencing fatigue. In addition, visual acuity tends to diminish with age.^38^

Our study used posters to deliver educational resources. Most diabetes self-management applications utilized in studies lacked any substantive educational content, with those that did offer such information providing only generic material. This finding is representative of the broader market landscape of available diabetes self-management applications, which generally fail to incorporate meaningful educational components. This is particularly noteworthy given that both clinical practice guidelines and the scholarly literature emphasize the critical role of education in enhancing motivation for self-management and promoting positive behavioral change.^39,40^

Our smartphone application demonstrates optimal scores on validation using PEMAT-A/V. The findings of this study are consistent with those reported in other investigations employing comparable methodologies, which have revealed that educational resources spanning diverse healthcare domains exhibit adequate validation metrics. The application leverages multimedia components, such as infographic flip charts, videos, and photos with descriptions, to deliver educational materials and exercise instructions to its target audience of older adults. This multimedia approach has been shown to facilitate information processing, enabling older individuals to comprehend abstract concepts better and retain information.^41,42^ Experts and end users rated the application highly for clarity and actionable guidance, suggesting effective communication.

### Strengths

The smartphone application was developed based on a thorough needs assessment. Second, the study had a multidisciplinary expert team, and the study took into consideration the needs of LMICs and geriatric-specific design principles. Third, the smartphone application had comprehensive education modules that were culturally relevant to LMICs. Lastly, the promising usability results and design elements tailored for geriatric populations suggest its potential for use in clinical trials.

### Limitations

The study had several limitations. First, the sample size was from a single geographic region. Second, while this study focused on usability and no intervention-related health outcomes were assessed, subsequent stages of the trial are set to rigorously evaluate the effectiveness of the intervention, specifically measuring its impact on crucial clinical outcomes.

### Implications for Future Research

Future research should include diverse populations to enhance generalizability, longitudinal follow-up to understand long-term impacts, and rigorous effectiveness evaluations to inform evidence-based practices. Further research is necessary to determine the smartphone application’s efficacy in enhancing clinical results and facilitating sustained self-care among various older adult demographics.

## Conclusion

A smartphone application was designed, developed, and validated and proven to be an effective, user-friendly rehabilitation tool designed specifically for older adults with T2DM in LMICs and is now ready to assess its effectiveness in enhancing the delivery of comprehensive rehabilitation programs for older adults with T2DM. Nevertheless, regular updates to the application remain crucial.

## Supplemental Material

sj-docx-1-dst-10.1177_19322968251365664 – Supplemental material for Smartphone Application-Based Rehabilitation Program for Older Adults With Type 2 Diabetes Mellitus: Insights From the Design, Development, and Validation Supplemental material, sj-docx-1-dst-10.1177_19322968251365664 for Smartphone Application-Based Rehabilitation Program for Older Adults With Type 2 Diabetes Mellitus: Insights From the Design, Development, and Validation by Tina Agnes, G. Arun Maiya, Muralidhar M. Kulakarni, Sawan Ramesh Hegde, Kirthinath Ballal and Mamatha Balachandra in Journal of Diabetes Science and Technology
