# Supplementary material for: Smartphone Application-Based Rehabilitation Program for Older Adults With Type 2 Diabetes Mellitus: Insights From the Design, Development, and Validation
Source: J Diabetes Sci Technol. 2025 Aug 25:19322968251365664. Online ahead of print. doi: 10.1177/19322968251365664 (PMC12380731; doi:10.1177/19322968251365664)
Supplement: sj-docx-1-dst-10.1177_19322968251365664 – Supplemental material for Smartphone Application-Based Rehabilitation Program for Older Adults With Type 2 Diabetes Mellitus: Insights From the Design, Development, and Validation [file sj-docx-1-dst-10.1177_19322968251365664.docx]

Supplementary file

Smartphone Application-based Rehabilitation Program for Older Adults with Type 2 Diabetes Mellitus - Insights from the Design, Development, and Validation

**Supplementary material No. 1**: Six modules of the application

**Supplementary material No. 2** Delivery of exercises week-wise


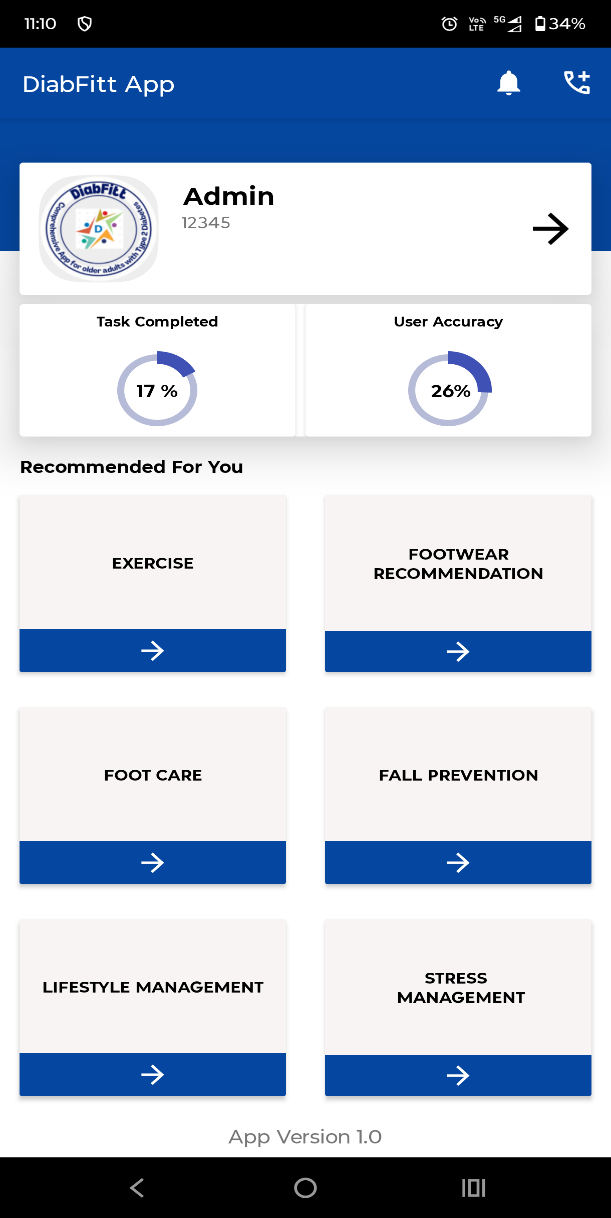

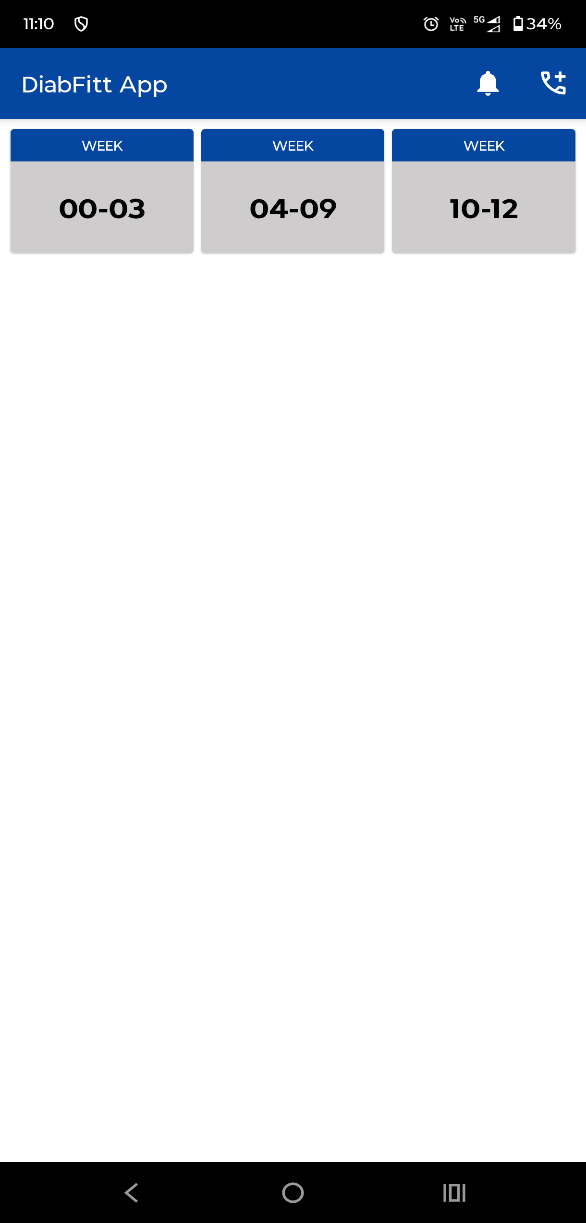


**Supplementary material No. 3:** Choice of language on the android application before login

**Supplementary material No. 4:** Entry of Five-digit verification code for login


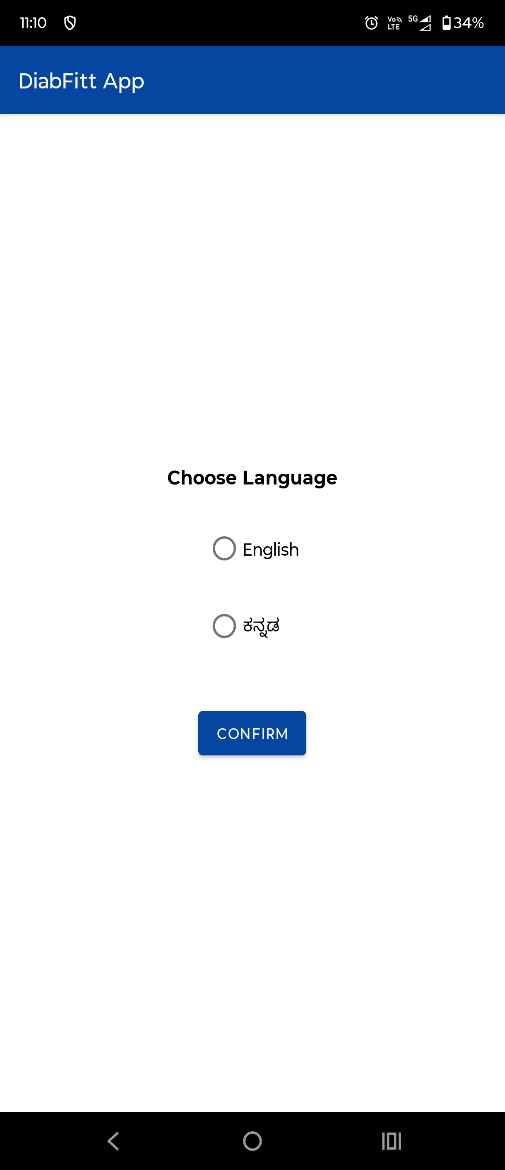

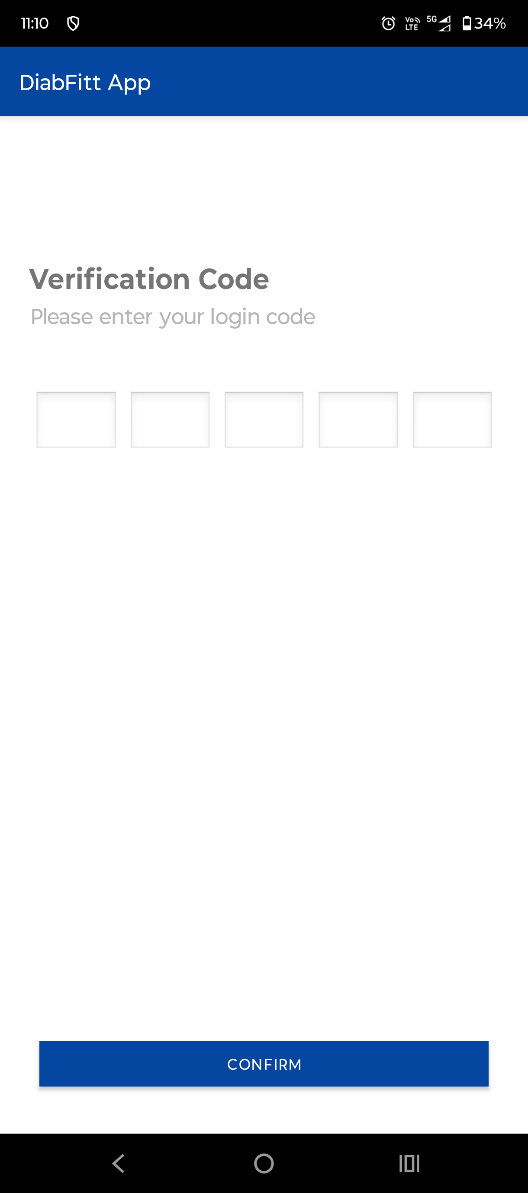


**Supplementary material No. 5:** Web Application to Participants


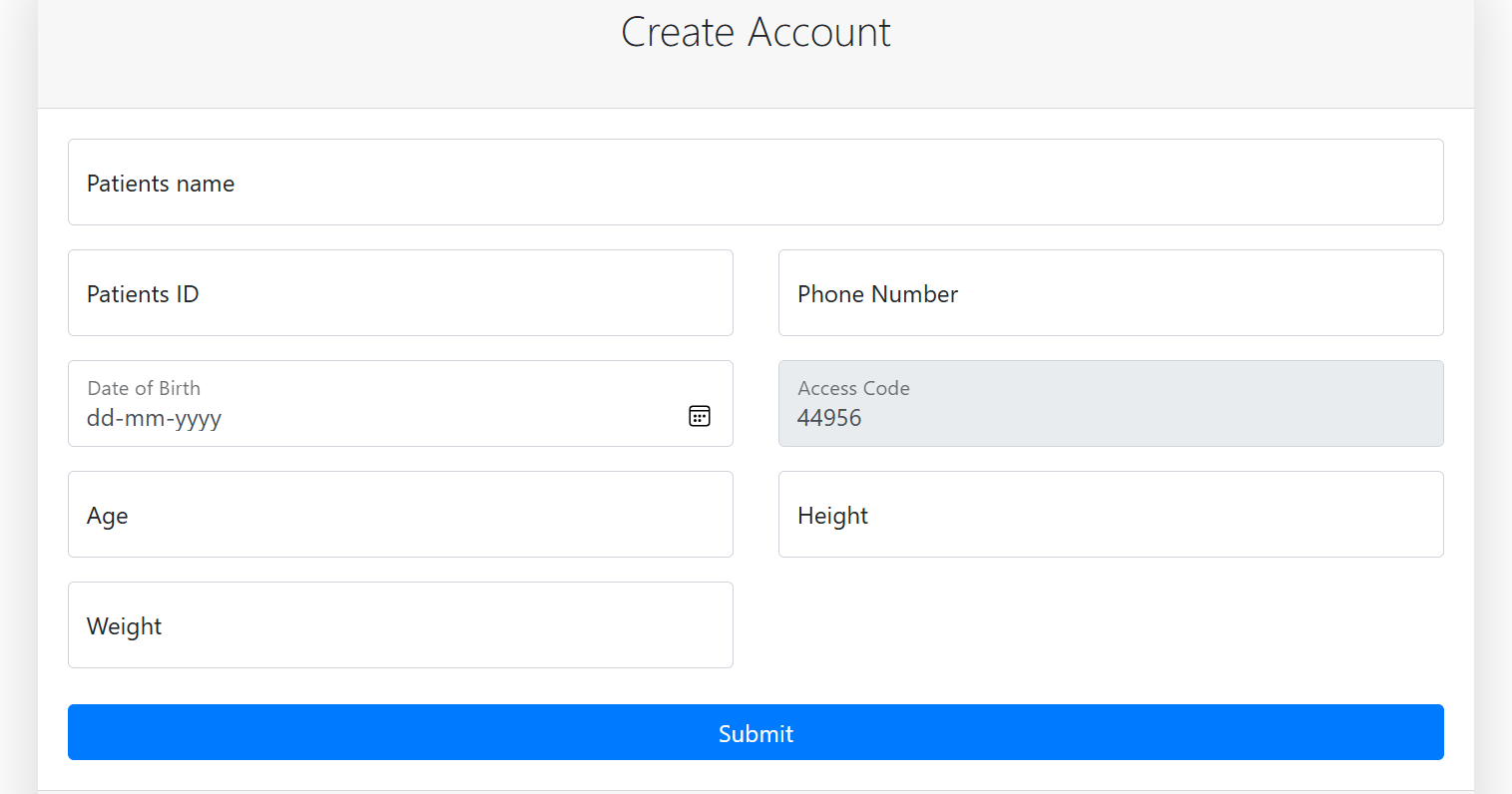


**Supplementary material No. 6:** Exercise delivery


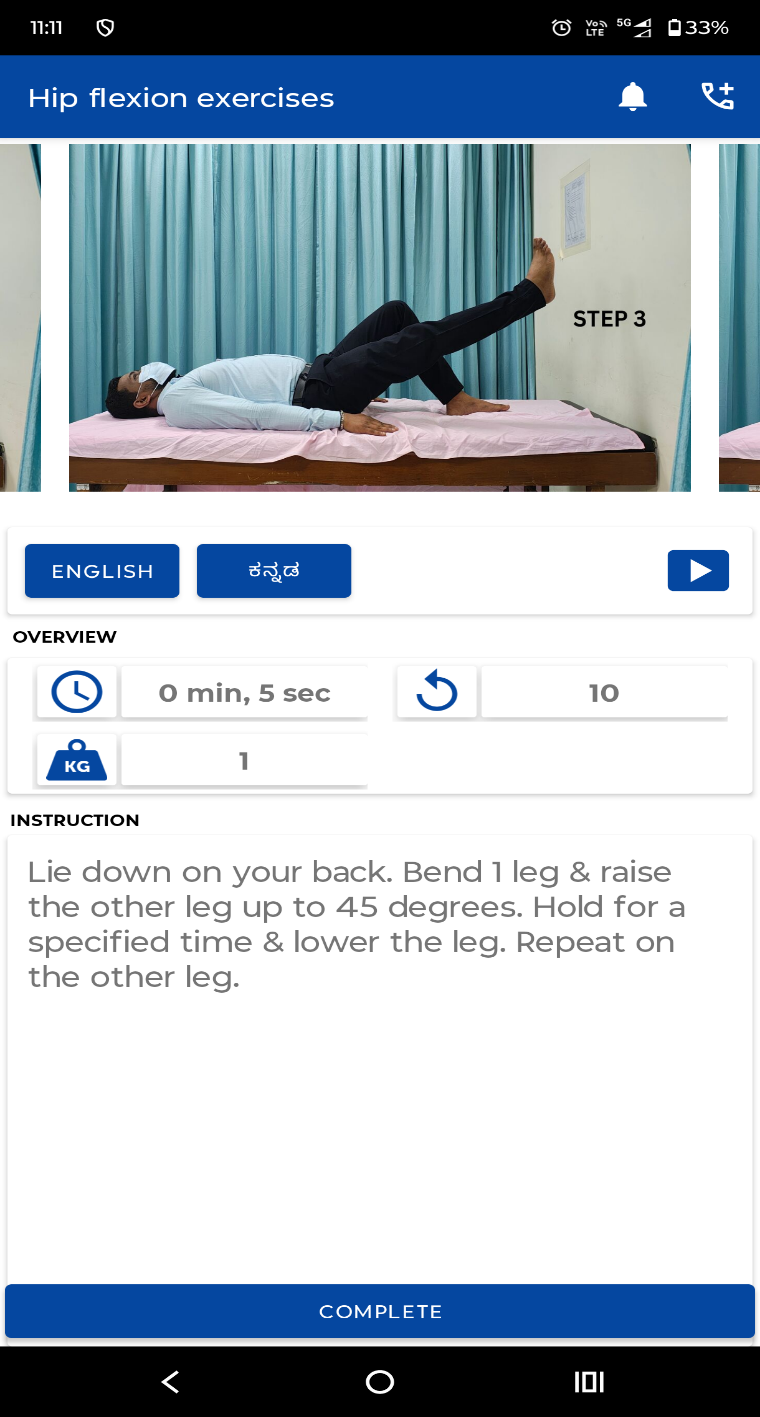


Web Application to Participants


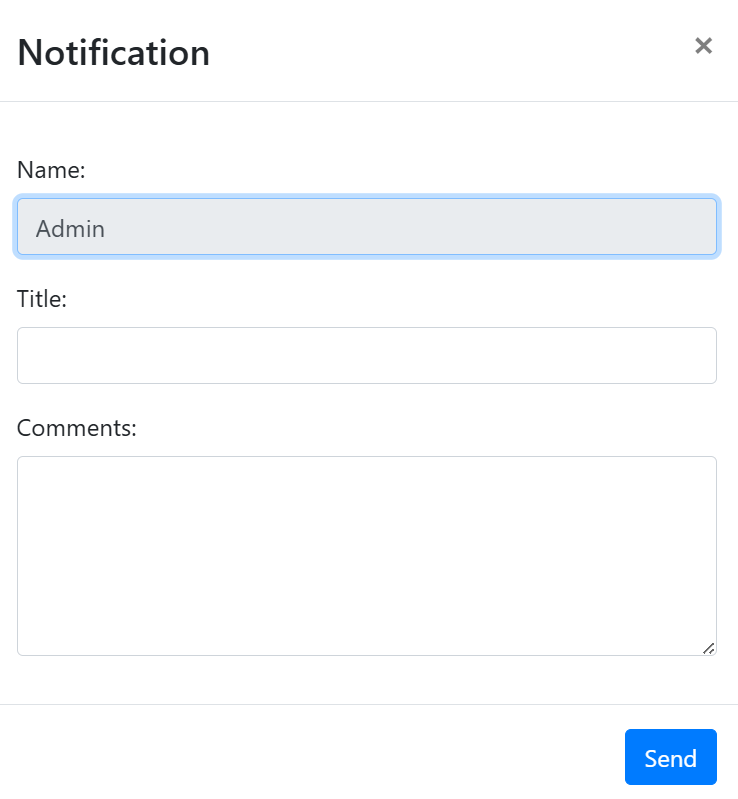


**Supplementary material No. 8:** Notification reminder


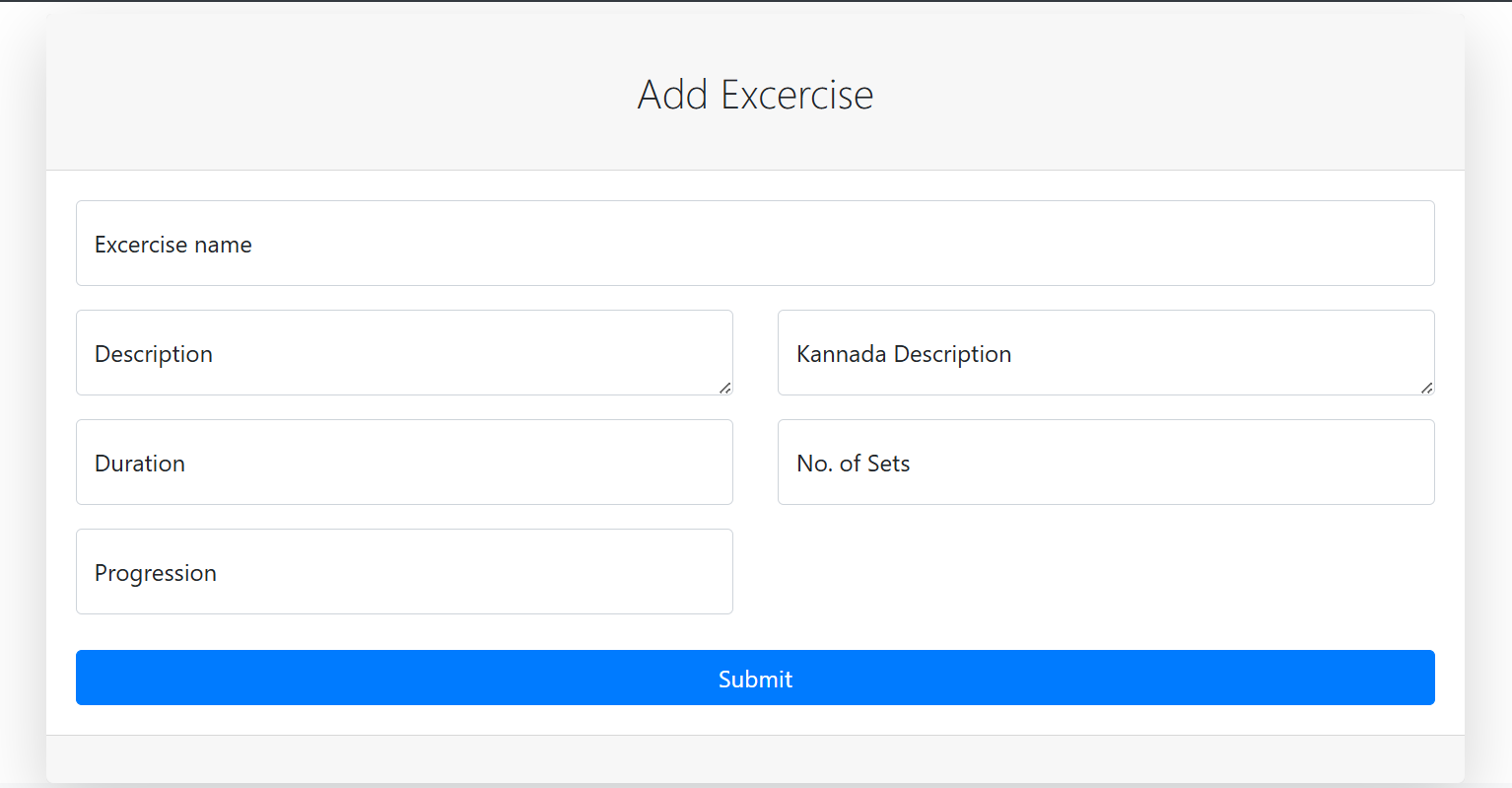


**Supplementary material No. 7:** Web Application to add exercises


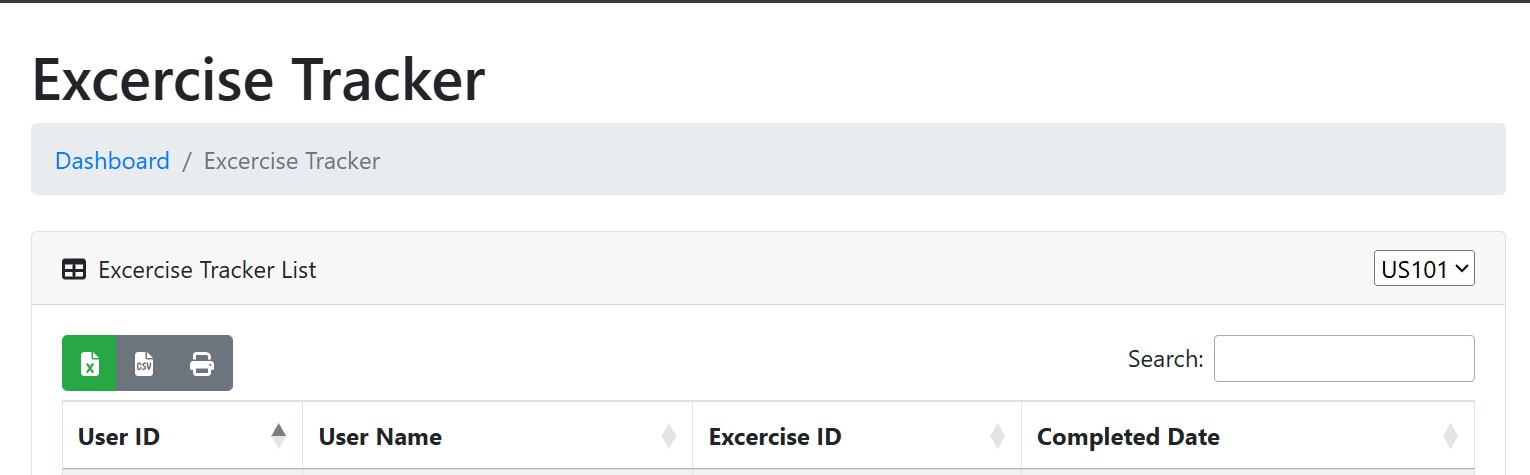


**Supplementary material No. 9:** Exercise tracker

Supplementary material No. 10: Progress monitoring
